# True Pulsatile Lumen Visualization in Coronary Artery Anomalies Using Controlled Transducer Pullback and Automated IVUS Segmentation

**DOI:** 10.1016/j.jaccas.2025.104741

**Published:** 2025-08-06

**Authors:** Anselm Walter Stark, Marius Reto Bigler, Lorenz Räber, Christoph Gräni

**Affiliations:** Inselspital, Bern University Hospital, University of Bern, Bern, Switzerland

**Figure:**
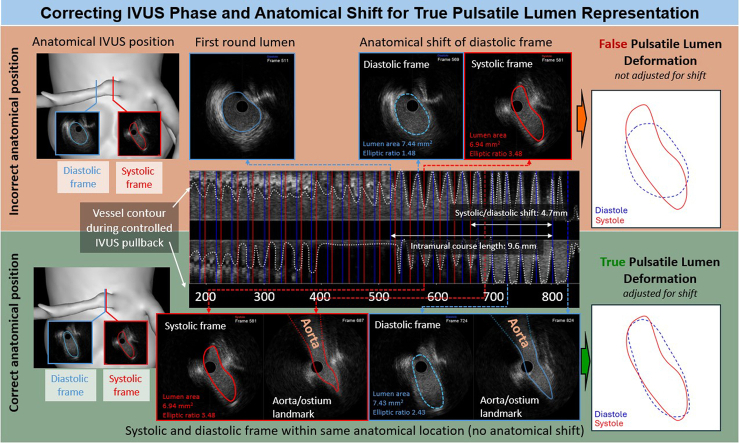

We read with great interest the case report by Yoshikawa et al^1^ describing the invasive evaluation of a 17-year-old patient with an anomalous aortic origin of a right coronary artery (R-AAOCA). Their use of invasive pressure measurements and intravascular ultrasound (IVUS) underscores the importance of assessing pulsatile lumen deformation during the cardiac cycle under resting and stress conditions in this clinical setting.

**Figure 1:**
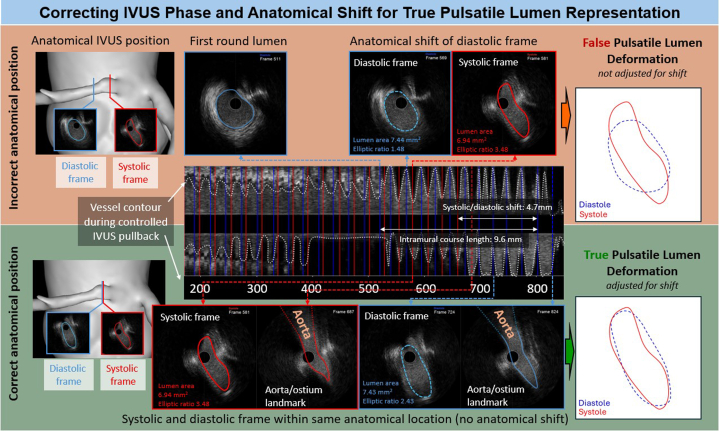
Comparison of “False” and “True” Pulsatile Lumen Deformation in the Ostium/Intramural Course of AAOCA, Highlighting the Benefit of IVUS Phase and Anatomical-Shift Correction

Here, we would like to comment on the IVUS imaging methodology. Stationary IVUS recordings at the ostium or intramural segment can show apparent “false pulsatile lumen deformation” between systole and diastole owing to catheter-induced anatomical shifts rather than actual vessel dynamics. This artifact, caused by relative coronary-to-transducer movement, results in the imaging plane shifting into or away from the ostium or intramural segment during the cardiac cycle. This may lead to the impression of exaggerated ostial or intramural lumen deformation when neighboring frames are directly compared. Although it does not affect the assessment of minimal lumen area, it can result in a false interpretation of systolic-diastolic lumen deformation at a specific anatomical location. Hence, a continuous, controlled, and standardized pullback at a low constant speed (eg, 1 mm/s) is recommended. Slower pullback increases frame density, allowing for more precise comparison of images taken at the same anatomical location in different cardiac phases. Spatial resolution along the catheter depends on both pullback speed and the patient's heart rate. Furthermore, postprocessing manual selection of systole and diastole based on electrocardiogram—although not feasible on all systems—or automated systole/diastole frame selection and matching at a fixed anatomical reference, can enhance analysis. One such tool, AIVUS-CAA, is a fully automated, open-source software program available on GitHub.^2^ Trained in vivo and validated in vitro using a flow-loop model, the AIVUS-CAA program segments vessel contours, computes cross-sectional areas, and aligns corresponding frames throughout the cardiac cycle. This corrects for apparent transducer motion artifacts and captures true pulsatile lumen changes (Figure 1).

Adopting a controlled pullback technique and correcting for IVUS phase and anatomical shift may improve diagnostic precision and inform more tailored treatment decisions in AAOCA.
